# CoAt-Set: Transformed coordinated attack dataset for collaborative intrusion detection simulation

**DOI:** 10.1016/j.dib.2025.111354

**Published:** 2025-02-03

**Authors:** Aulia Arif Wardana, Grzegorz Kołaczek, Parman Sukarno

**Affiliations:** a Wrocław University of Science and Technology, Poland; b Telkom University, Indonesia

**Keywords:** Cybersecurity, Anomaly detection, Network simulation, Augmented data, Heterogeneous data

## Abstract

The **CoAt-Set** dataset is a transformed dataset specifically designed for collaborative anomaly detection within Collaborative Intrusion Detection Systems (CIDS). It is developed by extracting and relabeling coordinated attack patterns from well-established datasets, including CIC-ToN-IoT, CIC-IDS2017, CIC-UNSW-NB15, CSE-CIC-IDS2018, CIC-BoT-IoT, Distrinet-CIC-IDS2017, and NF-UQ-NIDS. CoAt-Set focuses on coordinated attack scenarios such as large-scale stealthy scans, worm outbreaks, and distributed denial-of-service (DDoS) attacks, simulating realistic and high-impact threats that commonly observed in modern networks. The transformation process involved organizing coordinated attack behaviors and providing detailed annotations and network traffic features, enhancing its relevance for anomaly detection in collaborative environments. CoAt-Set is compatible with standard machine learning frameworks, offering researchers and practitioners a comprehensive resource for developing, testing, and evaluating CIDS models. It is suitable for various applications, including collective threat intelligence research, analyzing distributed threat patterns, developing machine learning algorithms for distributed systems, and training simulations designed for heterogeneous network environments.

Specifications Table

**Table:** 

Subject	Computer Sciences
Specific subject area	*Collaborative anomaly detection and network security analysis using dataset transformation for coordinated cyber-attack simulation*
Type of data	*Parquet Files*
Data collection	The CoAt-Set dataset was collected and curated from various well-established cybersecurity datasets, serving as the secondary data for this research. These include CIC-IDS2017, CSE-CIC-IDS2018, CIC-ToN-IoT, CIC-BoT-IoT, CIC-UNSW-NB15, and Distrinet-CIC-IDS2017. The extraction process focused on coordinated attack patterns, such as large-scale stealthy scans, worm outbreaks, and DDoS attacks, which are not comprehensively defined in the original datasets. By consolidating and transforming traffic patterns from these sources, the CoAt-Set introduces unique variables that make the study distinctive and highly relevant to collaborative intrusion detection in distributed environments. Python scripts and libraries such as Pandas and NumPy facilitated data manipulation and preprocessing, while data normalization was conducted to scale numerical features to a common range, ensuring compatibility and comparability. To enhance the dataset's quality, inclusion criteria focused on traffic relevant to coordinated attacks, excluding irrelevant entries. This thoughtful curation of secondary data ensures that the CoAt-Set dataset fills critical gaps in existing research and provides significant added value for the study of CIDS.
Data source location	*The CoAt-Set dataset was developed using secondary data collected from publicly available cybersecurity datasets. These include CIC-IDS2017, CIC-BoT-IoT, CIC-UNSW-NB15, CSE-CIC-IDS2018, Distrinet-CIC-IDS2017, and NF-UQ-NIDS, which served as foundational sources for this research. The datasets were chosen for their relevance in capturing diverse and realistic network traffic, enabling the extraction of coordinated attack patterns such as large-scale stealthy scans, worm outbreaks, and DDoS attacks. These secondary datasets, originally designed for generic intrusion detection, were transformed and augmented in CoAt-Set to focus specifically on CIDS and coordinated attacks. In particular, the inclusion of NF-UQ-NIDS, a dataset created from heterogeneous network traffic, enables the simulation of* Non-Independent and Identically Distributed *(Non-IID) scenarios, adding a novel dimension to this study. Since the datasets consist of synthetic network traffic rather than geographically linked data, geographical coordinates are not specified. By leveraging these secondary data sources, CoAt-Set provides a unique and comprehensive resource that significantly adds value to the study of coordinated attacks in distributed environments.*
Data accessibility	Repository name: CoAt-Set (Coordinated Attack Dataset) on Heterogeneous Computer Network Data identification number: 10.17632/28tmfg3rzb.2 Direct URL to data: https://data.mendeley.com/datasets/28tmfg3rzb/2 Instructions for accessing these data: A sample use of the dataset for CIDS based on Ensemble Learning and Federated Learning can be found at this link: 10.5281/zenodo.14279854
Related research article	*Wardana, A.A., Kołaczek, G., Warzyński, A. et al. Collaborative intrusion detection using weighted ensemble averaging deep neural network for coordinated attack detection in heterogeneous network. Int. J. Inf. Secur.****23****, 3329–3349 (2024)*. https://doi.org/10.1007/s10207-024-00891-3

## 1 Value of the Data

• The CoAt-Set dataset addresses the critical challenge of detecting coordinated attacks, such as stealthy scans, worm outbreaks, and DDoS attacks [1], which traditional Intrusion Detection Systems (IDS) often fail to identify. By focusing on complex, multi-network attack patterns, it enables researchers to develop and test innovative strategies for CIDS to protect heterogeneous networks effectively. CoAt-Set provides a standardized benchmark for evaluating machine learning and statistical methods in cybersecurity, facilitating consistent performance comparisons across diverse approaches. It supports advancements in anomaly detection mechanisms and inspires the exploration of novel algorithms for analyzing and interpreting network attack data.
• CoAt-Set enhances CIDS research by introducing unique features not found in existing IDS public datasets. It focuses on multiple datasets representing diverse network environments, enabling the simulation of multi-sensor scenarios to explore the integration of different sensors in CIDS. CoAt-Set enhances the representation of coordinated attacks by incorporating CIC-UNSW-NB15, which broadens traffic diversity, and Distrinet-CIC-IDS2017, addressing gaps in DDoS and scanning attack patterns found in datasets from research [2]. These additions provide a more comprehensive dataset for studying coordinated attacks. Furthermore, CoAt-Set introduces Non-IID scenarios by leveraging the unified NF-UQ-NIDS dataset, enabling researchers to simulate realistic heterogeneity in network traffic. Researchers also have the flexibility to choose between heterogeneous and distributed network simulations or Non-IID scenarios, offering greater versatility in studying complex attack behaviors.

## 2 Background

The motivation behind cyberattacks has shifted from seeking recognition to pursuing financial gain. Instead of enhancing their reputations through website defacements, many hackers now engage in activities such as spamming, phishing, and DDoS extortion to generate revenue. This transition reflects a broader trend where new vulnerabilities, often called zero-day exploits, are frequently traded within underground networks. Large-scale coordinated attacks, including stealthy scans, worm outbreaks, and DDoS attacks, have emerged as effective strategies for maximizing profits [2,3].

Attackers today leverage automation to exploit multiple vulnerabilities simultaneously, rather than targeting high-value assets one by one. The attack process often begins with stealthy scans to identify software vulnerabilities that may evade local IDS. Following these scans, attackers exploit known vulnerabilities to deploy worm scripts, compromising numerous hosts at once. These compromised systems, often called “zombies” or “bots,” are then used to execute DDoS attacks or sold on the dark web. By accumulating thousands of these compromised hosts, attackers can orchestrate large-scale DDoS campaigns to blackmail service providers for money [4].

To effectively study these complex attack behaviors, the CoAt-Set dataset has been developed using dataset transformation techniques, extracting and restructuring data from established real-world sources [5]. This approach enables researchers to create controlled environments where specific attack scenarios can be analyzed without the ethical and legal concerns associated with using sensitive, real-world attack data. Real-world datasets may contain confidential information or be subject to restrictions that limit accessibility, but through dataset transformation, CoAt-Set ensures compliance with data protection regulations and ethical standards while maintaining the integrity of attack patterns.

By incorporating diverse and comprehensive attack scenarios, the dataset allows researchers to explore a wide range of coordinated attacks, including those that are rare or emerging. The transformation process enhances the dataset's utility, providing a richer resource that supports the evaluation of detection algorithms under various conditions. This method significantly advances cybersecurity research by facilitating in-depth examinations of complex attack behaviors in a safe, manageable context. The **CoAt-Set** dataset helps researchers develop robust detection strategies within CIDS, enhancing our understanding of coordinated attacks and contributing to improved cybersecurity defenses.

## 3 Data description

### 3.1 General information

The CoAt-Set dataset builds upon and significantly enhances the coordinated attack dataset introduced in the research [2]. While the previous research utilized datasets such as CIC-IDS2017, CSE-CIC-IDS2018, CIC-ToN-IoT, and CIC-BoT-IoT to simulate coordinated attacks, CoAt-Set introduces several key advancements. First, CoAt-Set includes CIC-UNSW-NB15 as a new network source, broadening the diversity of traffic patterns and coordinated attack scenarios. Additionally, Distrinet-CIC-IDS2017 is incorporated as a feeder dataset to address specific gaps in coordinated attack patterns, such as the lack of DDoS traffic in CIC-UNSW-NB15 and incomplete scanning attack patterns in CSE-CIC-IDS2018. These additions ensure that CoAt-Set offers a more comprehensive and balanced dataset for studying coordinated attacks.

Second, CoAt-Set introduces a Non-IID simulation scenario, a significant advancement over the research [2]. This scenario leverages the NF-UQ-NIDS dataset, which has been transformed and relabeled during the CoAt-Set builder process. NF-UQ-NIDS is a unified dataset created from multiple public sources, specifically designed to represent heterogeneous network traffic. It contains diverse patterns of coordinated attacks, making it uniquely suited for simulating Non-IID scenarios. By including NF-UQ-NIDS, researchers can study scenarios involving heterogeneity and distribution in network traffic, enhancing the realism and applicability of their simulations.

The CoAt-Set dataset was developed using a systematic transformation process that extracts and re-labels coordinated attack patterns from multiple well-established cybersecurity datasets, such as CIC-IDS2017, CIC-BoT-IoT, CIC-ToN-IoT, CIC-UNSW-NB15, CSE-CIC-IDS2018, Distrinet-CIC-IDS2017, and NF-UQ-NIDS. This transformation introduces unique variables that make the study distinctive, including a specific focus on coordinated attack scenarios like large-scale stealthy scans, worm outbreaks, and DDoS attacks. These variables are not explicitly defined in the original datasets, as they were primarily designed for generic intrusion detection rather than collaborative anomaly detection in distributed environments. By consolidating, cleaning, and harmonizing data from these sources, the CoAt-Set dataset is tailored to support simulations in heterogeneous network environments. Its structure facilitates the study of distributed attack behaviors across diverse network types, making it particularly suitable for evaluating the performance of CIDS under realistic and high-impact threat scenarios. The resulting dataset bridges existing gaps in intrusion detection research and provides a comprehensive resource for researchers, thereby aligning with the requirement to create substantial original value from secondary data.

The details of CoAt-Set dataset can be seen on Table 1. The dataset consists of five files that utilize CICFlowMeter features and one file that utilizes NetFlow features, each designed for distinct simulation purposes in CIDS. Specifically, the five CICFlowMeter-based files include datasets such as CoAt_CIC-BoT-IoT-V2, CoAt_CIC-IDS2017-V2, CoAt_CIC-ToN-IoT-V2, CoAt_CIC-UNSW-NB15_Feeded-V2, and CoAt_CSE-CIC-IDS2018_Feeded, all aimed at simulating multi-sensor CIDS in heterogeneous network environments. By maintaining separate files for different network sources, this dataset allows researchers to model diverse network traffic, reflecting the complexity of real-world, distributed networks. This structure is particularly beneficial for studying how coordinated attacks unfold across various network types and helps simulate traffic patterns from multiple sources effectively.

**Table 1:** List of dataset files, including details on the number of traffic records, file size, and dataset source.

Dataset (.parquet)	Number of Traffic				File Size (MB)	Source of Dataset
	Benign	Scan	Worm	DDoS		
CoAt_CIC-IDS2017-V2	1977318	1956	1473	128014	257.4	CIC-IDS2017
CoAt_CIC-BoT-IoT-V2	38435	1896036	801	4786502	896.88	CIC-BoT-IoT
CoAt_CIC-ToN-IoT-V2	2123867	35564	27145	202	180	CIC-ToN-IoT
CoAt_CIC-UNSW-NB15_Feeded-V2	3450658	46733	33751	95144	325.14	CIC-UNSW-NB15 Distrinet-CIC-IDS2017
CoAt_CSE-CIC-IDS2018_Feeded	5329008	1683	263018	775955	746.65	CSE-CIC-IDS2018 Distrinet-CIC-IDS2017
CoAt_NF-UQ-NIDS-V2	20743823	17302868	5397772	192134	1330	NF-UQ-NIDS

The CoAt_NF-UQ-NIDS-V2 file, based on NetFlow features and aggregating traffic from multiple datasets, is primarily intended for simulating coordinated attacks in more uniform, homogeneous networks. However, this dataset offers enough variety to be employed in heterogeneous network simulations as well, especially when using Non-IID data settings. This adaptability allows researchers to explore a wide range of coordinated attack scenarios, making the dataset a powerful tool for both straightforward homogeneous simulations and more complex heterogeneous network studies in the context of CIDS research.

### 3.2 Feature and type of data

The CoAt-Set dataset integrates features from CICFlowMeter and NetFlow, offering a comprehensive foundation for CIDS research. Details of the CICFlowMeter features used in this research are provided in Table 2, with feature descriptions accessible via the website [6]. These features enable in-depth statistical analysis of network traffic, capturing critical attributes such as packet length, flow duration, and protocol-specific metrics. Such detailed insights are essential for detecting complex attack behaviors in heterogeneous networks and supporting machine learning models by distinguishing between normal and malicious traffic patterns [7].

**Table 2:** CICFlowMeter feature and data type in CoAt-Set.

No	Feature Name	Data Type	No	Feature Name	Data Type
0	Protocol	int8	40	Packet Length Mean	float32
1	Flow Duration	int64	41	Packet Length Std	float32
2	Total Fwd Packets	int32	42	Packet Length Variance	float32
3	Total Backward Packets	int32	43	FIN Flag Count	int8
4	Fwd Packets Length Total	float64	44	SYN Flag Count	int8
5	Bwd Packets Length Total	float64	45	RST Flag Count	int16
6	Fwd Packet Length Max	float64	46	PSH Flag Count	int16
7	Fwd Packet Length Min	float32	47	ACK Flag Count	int32
8	Fwd Packet Length Mean	float32	48	URG Flag Count	int8
9	Fwd Packet Length Std	float32	49	CWE Flag Count	int8
10	Bwd Packet Length Max	float64	50	ECE Flag Count	int8
11	Bwd Packet Length Min	float32	51	Down/Up Ratio	float32
12	Bwd Packet Length Mean	float32	52	Avg Packet Size	float32
13	Bwd Packet Length Std	float32	53	Avg Fwd Segment Size	float32
14	Flow Bytes/s	float64	54	Avg Bwd Segment Size	float32
15	Flow Packets/s	float64	55	Fwd Avg Bytes/Bulk	int32
16	Flow IAT Mean	float32	56	Fwd Avg Packets/Bulk	int16
17	Flow IAT Std	float32	57	Fwd Avg Bulk Rate	int32
18	Flow IAT Max	float64	58	Bwd Avg Bytes/Bulk	int32
19	Flow IAT Min	float64	59	Bwd Avg Packets/Bulk	int32
20	Fwd IAT Total	float64	60	Bwd Avg Bulk Rate	int32
21	Fwd IAT Mean	float32	61	Subflow Fwd Packets	int32
22	Fwd IAT Std	float32	62	Subflow Fwd Bytes	int32
23	Fwd IAT Max	float64	63	Subflow Bwd Packets	int32
24	Fwd IAT Min	float64	64	Subflow Bwd Bytes	int32
25	Bwd IAT Total	float64	65	Init Fwd Win Bytes	int32
26	Bwd IAT Mean	float32	66	Init Bwd Win Bytes	int32
27	Bwd IAT Std	float32	67	Fwd Act Data Packets	int32
28	Bwd IAT Max	float64	68	Fwd Seg Size Min	int8
29	Bwd IAT Min	float64	69	Active Mean	float32
30	Fwd PSH Flags	int16	70	Active Std	float32
31	Bwd PSH Flags	int16	71	Active Max	float64
32	Fwd URG Flags	int8	72	Active Min	float64
33	Bwd URG Flags	int8	73	Idle Mean	float32
34	Fwd Header Length	int32	74	Idle Std	float32
35	Bwd Header Length	int32	75	Idle Max	float64
36	Fwd Packets/s	float32	76	Idle Min	float64
37	Bwd Packets/s	float32	77	Label	object
38	Packet Length Min	float32	78	Attack	int64
39	Packet Length Max	float64			

Similarly, details of the NetFlow features used in this research are presented in Table 3, with feature descriptions outlined in research [8]. NetFlow features focus on high-level communication behavior, such as packet flow and traffic frequency, offering lightweight and scalable metrics. This makes them particularly effective for monitoring large-scale networks and identifying distributed and coordinated attacks like DDoS [8].

**Table 3:** NetFlow feature and data type in CoAt-Set.

No	Feature Name	Data Type	No	Feature Name	Data Type
0	L4_SRC_PORT	int16	22	RETRANSMITTED_IN_BYTES	int32
1	L4_DST_PORT	int16	23	RETRANSMITTED_IN_PKTS	int16
2	PROTOCOL	int16	24	RETRANSMITTED_OUT_BYTES	int32
3	L7_PROTO	float32	25	RETRANSMITTED_OUT_PKTS	int16
4	IN_BYTES	int32	26	SRC_TO_DST_AVG_THROUGHPUT	int64
5	IN_PKTS	int32	27	DST_TO_SRC_AVG_THROUGHPUT	int64
6	OUT_BYTES	int32	28	NUM_PKTS_UP_TO_128_BYTES	int32
7	OUT_PKTS	int32	29	NUM_PKTS_128_TO_256_BYTES	int32
8	TCP_FLAGS	int16	30	NUM_PKTS_256_TO_512_BYTES	int32
9	CLIENT_TCP_FLAGS	int16	31	NUM_PKTS_512_TO_1024_BYTES	int32
10	SERVER_TCP_FLAGS	int16	32	NUM_PKTS_1024_TO_1514_BYTES	int32
11	DNS_TTL_ANSWER	int32	33	TCP_WIN_MAX_IN	int32
12	DURATION_IN	int32	34	TCP_WIN_MAX_OUT	int32
13	DURATION_OUT	int32	35	SRC_TO_DST_SECOND_BYTES	float32
14	MIN_TTL	int16	36	DST_TO_SRC_SECOND_BYTES	float32
15	MAX_TTL	int16	37	DNS_QUERY_ID	int32
16	LONGEST_FLOW_PKT	int32	38	DNS_QUERY_TYPE	int32
17	SHORTEST_FLOW_PKT	int16	39	FLOW_DURATION_MILLISECONDS	int32
18	MIN_IP_PKT_LEN	int16	40	FTP_COMMAND_RET_CODE	float32
19	MAX_IP_PKT_LEN	int32	41	Label	object
20	ICMP_TYPE	int32	42	Attack	int8
21	ICMP_IPV4_TYPE	int16			

The detailed descriptions of the features in Tables 2 and 3 are available in the dataset folder within the repository. By combining CICFlowMeter's detailed traffic analysis with NetFlow's scalability, the CoAt-Set dataset provides a versatile resource for CIDS applications. It supports tasks ranging from detailed anomaly detection to real-time network surveillance, making it suitable for both homogeneous and heterogeneous simulation scenarios. This adaptability ensures its relevance across diverse research contexts.

### 3.3 Data format

The dataset consists of tabular data organized into multiple files, each containing a huge volume of information. During the preprocessing step, the data format for all files is Comma-Separated Values (CSV). The CSV files are loaded using the Pandas library with the “pandas.read_csv” function. After preprocessing, the data is converted to the Parquet file format, which is specifically designed to facilitate efficient data processing and retrieval. To open and work with the Parquet files in the CoAt-Set dataset, researchers can use Python with Jupyter Notebook, leveraging the powerful Pandas library. The dataset files are stored in Parquet format to ensure efficient data storage and retrieval. To access the data, the “pandas.read_parquet” function can be used with the pyarrow engine. This combination allows for seamless reading of the dataset into a Pandas DataFrame for analysis and preprocessing. The sample code can be seen here: 10.5281/zenodo.14279854.

### 3.4 Applications and uses of the CoAt-Set dataset

The CoAt-Set dataset is designed to support a wide range of research applications in CIDS. Its unique features provide researchers with the flexibility to simulate diverse scenarios and address complex challenges in cybersecurity. Key applications include:

• **Heterogeneous and Distributed Network Simulation:** CoAt-Set facilitates the simulation of distributed network environments by utilizing multiple datasets with CICFlowMeter features. This enables researchers to create scenarios with diverse traffic patterns across multiple networks, mimicking real-world heterogeneous environments. Such simulations are invaluable for studying the effectiveness of CIDS in detecting coordinated attacks that span across different network segments.
• **Non-IID Scenario Simulation:** The dataset incorporates the unified NF-UQ-NIDS dataset, which includes NetFlow features, to simulate Non-IID scenarios. These scenarios are particularly useful for studying heterogeneous network traffic where distributions vary significantly between nodes. Researchers can use this feature to analyze the impact of data distribution heterogeneity on detection algorithms and develop strategies to enhance their performance in real-world applications.
• **Advancing CIDS Research:** By providing two distinct simulation strategies—heterogeneous and distributed network simulation, and Non-IID scenario simulation—CoAt-Set bridges gaps in existing research. It enables the study of complex attack behaviors in distributed environments, helping researchers develop and test innovative algorithms for anomaly detection, attack mitigation, and collaborative defense mechanisms.

These features make CoAt-Set a versatile and powerful resource, allowing researchers to address critical challenges in cybersecurity and advance the development of robust CIDS capable of handling the evolving landscape of coordinated attacks.

## 4 Experimental design, materials and methods

### 4.1 Data sources and experimental environment

The investigation was conducted using a server equipped with a 2.3 GHz 16-Core Intel(R) Xeon(R) CPU E5-2650 v3 and 128 GB of memory, running Ubuntu 22.04.2 LTS. The analysis relied on Python version 3.12.5, using Pandas version 2.2.3 and the pyarrow engine for efficient reading of Parquet files. Table 4 outlines the sources of datasets used in the creation of the CoAt-Set dataset, which includes key research initiatives like CIC-IDS2017, CIC-BoT-IoT, CIC-ToN-IoT, CIC-UNSW-NB15, CSE-CIC-IDS2018, Distrinet-CIC-IDS2017, and NF-UQ-NIDS. These datasets provide a robust foundation for studying network traffic and offer valuable insights into intrusion detection and network security. All datasets in Table 4 were selected because they contain traffic patterns relevant to coordinated attacks.

**Table 4:** Source of dataset.

Research	Dataset	Link
[7]	CIC-IDS2017	https://www.unb.ca/cic/datasets/ids-2017.html
[9]	CIC-BoT-IoT	https://staff.itee.uq.edu.au/marius/NIDS_datasets/#RA14
[9]	CIC-ToN-IoT	https://staff.itee.uq.edu.au/marius/NIDS_datasets/#RA13
[10]	CIC-UNSW-NB15	https://www.unb.ca/cic/datasets/cic-unsw-nb15.html
[6] [7]	CSE-CIC-IDS2018	https://www.unb.ca/cic/datasets/ids-2018.html
[8]	NF-UQ-NIDS	https://staff.itee.uq.edu.au/marius/NIDS_datasets/#RA10
[11]	Distrinet-CIC-IDS2017	https://intrusion-detection.distrinet-research.be/WTMC2021/tools_datasets.html

**Fig. 1:**
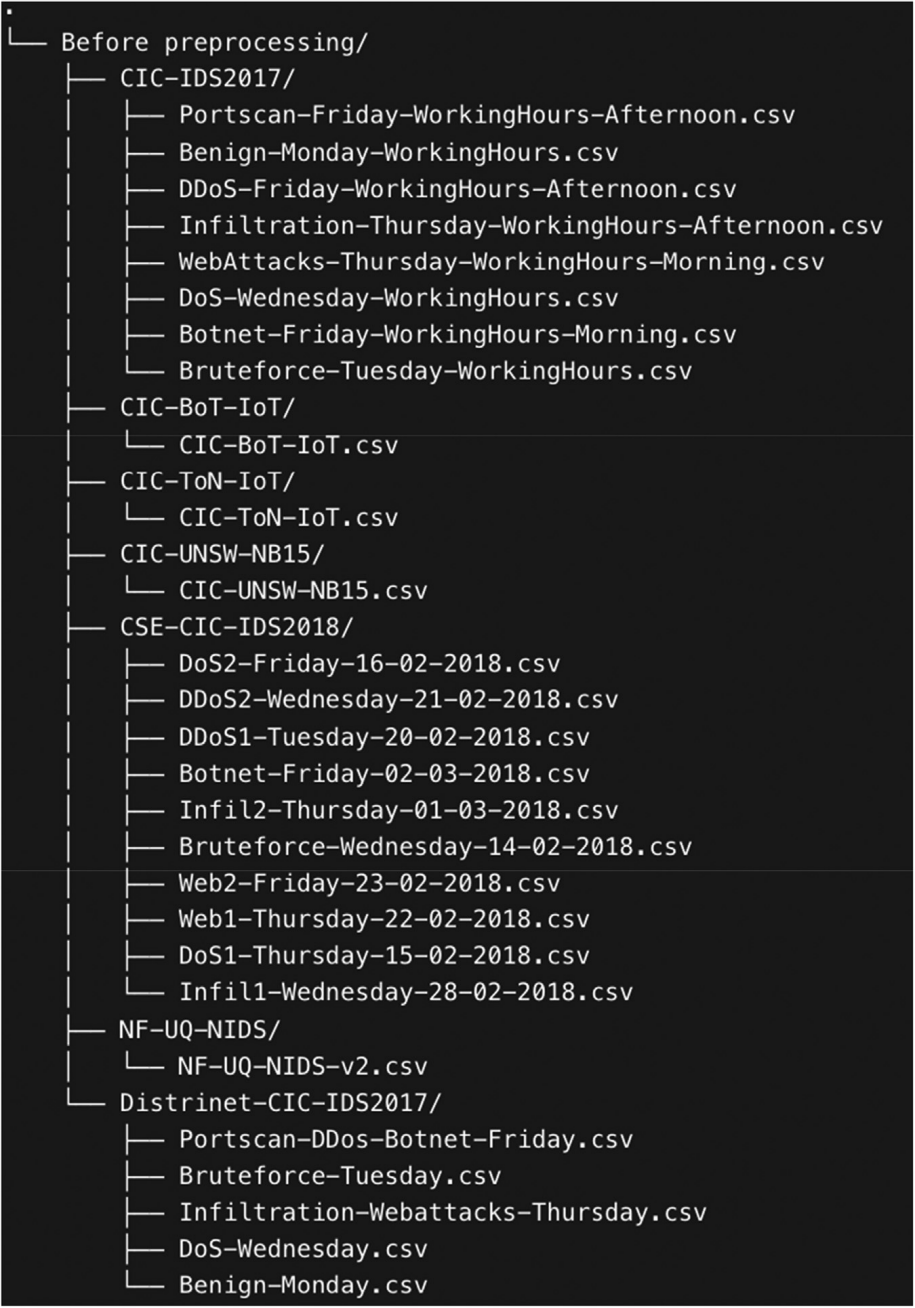
Raw data folder snapshot.

The datasets in Table 4 are systematically organized, as illustrated in Fig. 1, prior to preprocessing. The CIC-IDS2017, CSE-CIC-IDS2018, and Distrinet-CIC-IDS2017 datasets are categorized by attack types and temporal segments. The inclusion of both normal and malicious traffic ensures a balanced and realistic representation of network conditions, which is essential for the development and evaluation of machine learning models for IDS. The datasets shown in Fig. 1 will proceed through a preprocessing workflow that involves converting them from CSV to Parquet format, leveraging Parquet's columnar storage structure to achieve reduced file sizes and faster processing. Following the conversion, the datasets are cleaned and standardized through feature mapping to resolve inconsistencies. The CICFlowMeter-based feature datasets are stored in the folders CIC-IDS2017, CIC-BoT-IoT, CIC-ToN-IoT, CIC-UNSW-NB15, and Distrinet-CIC-IDS2017. The NetFlow-based feature dataset is stored in the folder NF-UQ-NIDS.

### 4.2 Data Preprocessing

The preprocessing dataset outlined in Algorithm 1 provides a structured and systematic approach to transforming raw data into clean and efficient Parquet files, making them suitable for the data consolidation step in the CoAt-Set process before re-labeling the data. The preprocessing algorithm in Algorithm 1 is used for processing the CIC-IDS2017, CIC-BoT-IoT, CIC-ToN-IoT, CIC-UNSW-NB15, CSE-CIC-IDS2018, NF-UQ-NIDS, and Distrinet-CIC-IDS2017 datasets (line 1). The output from the processed data is a dataset that is ready to be used for the data consolidation step in the data transformation process, in Parquet file format (line 2).

**Table:** 

**Algorithm 1**: Preprocessing dataset.

1: **Input**: List of raw CSV files *F = {f_1_, f_2_, . . ., f_n_}*. 2: **Output**: Cleaned Parquet file *P = {p_1_, p_2_, . . ., p_n_}*. 3: **Step 1: Load Data** 4: **for** each file *f_i_ ∈ F***do** 5: Load the CSV file into a DataFrame *D_i_*. 6: **end for** 7: **Step 2: Remove Irrelevant Columns** 8: **for** each DataFrame *D_i_***do** 9: **if***D_i_* uses CICFlowMeter features **then** 10: Identify columns to include: *C_include_* = {all features in Table 2}. 11: **else** 12: Identify columns to include: *C_include_* = {all features in Table 3}. 13: **end if** 14: Remove all features except *C_include_* from *D_i_*. 15: **end for** 16: **Step 3: Standardize Column Names** 17: **for** each DataFrame *D_i_***do** 18: Strip whitespace from column names in *D_i_*. 19: Rename columns based on a predefined mapping in *C_include_* for consistency. 20: **end for** 21: **Step 4: Clean Labels** 22: **for** each DataFrame *D_i_***do** 23: Replace inconsistent label values (e.g., replace BENIGN with Benign). 24: **end for** 25: **Step 5: Handle Missing and Infinite Values** 26: **for** each DataFrame *D_i_***do** 27: Replace infinite values (∞, −∞) with NaN. 28: Remove rows containing NaN values. 29: **end for** 30: **Step 6: Remove Duplicate Rows** 31: **for** each DataFrame *D_i_***do** 32: Identify and remove duplicate rows. 33: Reset the index of *D_i_*. 34: **end for** 35: **Step 7: Save Cleaned Data** 36: **for** each DataFrame *D_i_***do** 37: Save *D_i_* as a Parquet file *p_i_*. 38: **end for** 39: **Return**: The set of Parquet files *P = {p_1_, p_2_, . . ., p_n_}*.

The preprocessing journey begins with loading the raw datasets stored as CSV files. Each file represents network data containing numerous features and target labels. This step involves reading these files into a data manipulation tool such as pandas to create DataFrames. These DataFrames act as a structured representation of the CSV data (line 3-6). Datasets often include columns that do not contribute to the analysis or may introduce noise. We utilized the features listed in Table 2 for datasets such as CIC-IDS2017, CIC-BoT-IoT, CIC-ToN-IoT, CIC-UNSW-NB15, CSE-CIC-IDS2018, and Distrinet-CIC-IDS2017. For NF-UQ-NIDS, we relied on the features outlined in Table 3. These features serve as the primary attributes in the CoAt-Set dataset (line 7-15).

Inconsistent naming conventions across datasets can lead to errors and inefficiencies during data analysis. Standardizing column names ensures uniformity and eliminates ambiguity. This step includes stripping unnecessary whitespace, renaming columns based on feature mapping in Tables 2 and 3. Consistent naming conventions also facilitate merging or comparing multiple datasets in later stages (line 16-20). The target column (Label) often contains inconsistencies such as variations in casing (e.g., replace BENIGN with Benign) or minor typographical differences. Normalizing the labels ensures that all categories are consistent and suitable for downstream tasks like classification (line 21-24).

Missing and infinite values pose significant challenges to data quality. Infinite values may arise from erroneous calculations, while missing values often result from incomplete data collection. Identifying and replacing infinite values with Not a Number (NaN) ensures compatibility with data-cleaning functions, and rows containing missing values are subsequently removed to maintain dataset integrity. In addition, duplicate rows in the dataset are redundant and can distort statistical analyses or machine learning models. Detecting and removing these duplicates reduces the size of the dataset while preserving its essential characteristics. After eliminating duplicates, resetting the DataFrame index ensures that the dataset maintains a coherent structure and facilitates smooth operations in subsequent steps (line 25-34). After preprocessing, the cleaned datasets are saved in the Parquet format (line 35-39).

### 4.3 Transformation data process

**Fig. 2:**
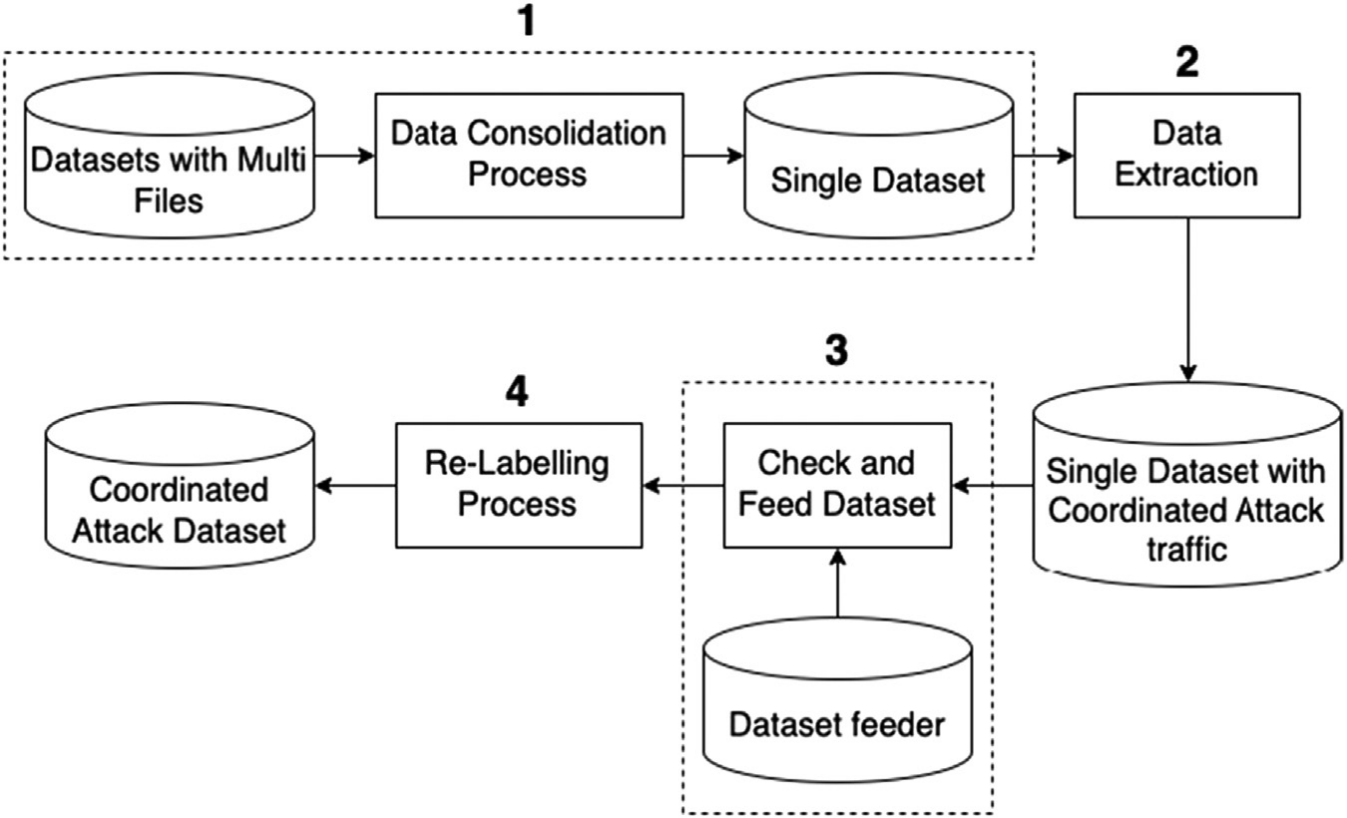
Data transformation process.

Fig. 2 represents a data transformation process workflow designed to prepare a dataset containing coordinated attack traffic. The process begins with multiple datasets that are combined into a single, unified dataset through a **Data Consolidation** process. The Data Consolidation process is applicable only to datasets with multiple files. If a dataset consists of a single file, this step is skipped. This step ensures that all data is standardized and manageable. Once the data is consolidated, a **Data Extraction** phase takes place, where specific information is extracted from the unified dataset to include features relevant to identifying coordinated attack traffic. The result is a single dataset enriched with normal and coordinated attack traffic data.

Next, the **Data Checking** process ensures that the dataset contains a complete set of coordinated attack traffic patterns, such as large-scale stealthy scans, worm outbreaks, and DDoS attacks. If any of these patterns are missing, we supplement the dataset by incorporating the missing coordinated attack traffic from another dataset that contains the required patterns. The dataset providing the missing patterns is referred to as the “dataset feeder.” Once all datasets have a complete set of coordinated attack patterns, they proceed to the **Data Re-Labelling** process. In this step, the data is categorized and labelled based on the characteristics of coordinated attacks. The labelling is performed through a thorough analysis of typical traffic patterns associated with coordinated attacks, which are then grouped into similar attack categories. The final result is a refined and labelled dataset that specifically highlights coordinated attack traffic, saved in Parquet file format.

**Fig. 3:**
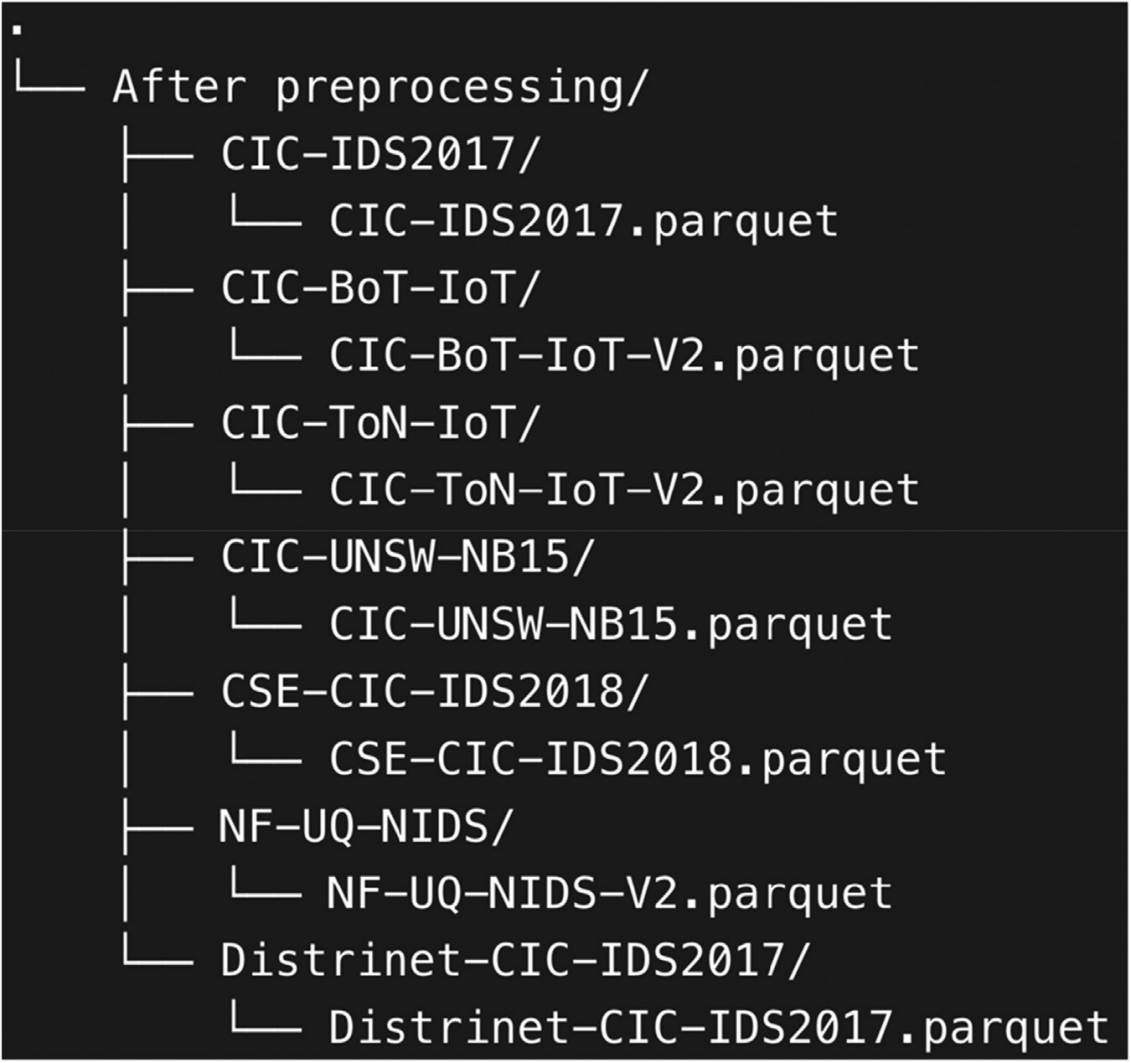
Data consolidation result.

**Data Consolidation**: This process involves merging datasets that contain multiple files into a single, unified dataset. Datasets such as CIC-IDS2017, CSE-CIC-IDS2018, and Distrinet-CIC-IDS2017, which are originally divided into multiple files, are consolidated into one comprehensive dataset. This step is essential for standardizing and organizing the data, making it more manageable and suitable for subsequent processing stages. The outcome of this data consolidation process is illustrated in Fig. 3, providing a clear representation of how each files in CIC-IDS2017, CSE-CIC-IDS2018, and Distrinet-CIC-IDS2017 are combined into a single dataset.

**Data Extraction, Checking, and Re-Labelling Process**: The relabeling process involved grouping certain attacks that are associated with coordinated attack patterns. Details on the traffic extracted from the source datasets and the relabeling process can be found in Table 5. The traffic related with nomal traffic and coordinated attack traffic from each dataset is extract like in column “Label of extracted data”. Two datasets, CIC-UNSW-NB15 and CSE-CIC-IDS2018, do not have complete coordinated attack patterns. CIC-UNSW-NB15 lacks DDoS attack patterns, and CSE-CIC-IDS2018 is missing scanning attack patterns. To address these gaps, we supplemented these two datasets with traffic from the Distrinet-CIC-IDS2017 dataset. While similar to CIC-IDS2017, Distrinet-CIC-IDS2017 includes some improvements and variations. We chose this dataset to introduce diversity into our data by incorporating insights from other research. Distrinet-CIC-IDS2017 retains most of the traffic patterns from CIC-IDS2017, making it highly suitable for capturing coordinated attack traffic.

**Table 5:** Re-Labelling in CoAt-Set dataset.

CoAt-Set File	Dataset Source	Label of extracted data	New Label
CoAt_CIC-IDS2017-V2	CIC-IDS2017	PortScan	Scan
		Infiltration, Bot	Worm
		DDoS	DDoS
		Benign	Benign
CoAt_CIC-BoT-IoT-V2	CIC-BoT-IoT	Reconnaissance	Scan
		Theft	Worm
		DDoS	DDoS
		Benign	Benign
CoAt_CIC-ToN-IoT-V2	CIC-ToN-IoT	scanning	Scan
		backdoor	Worm
		ddos	DDoS
		Benign	Benign
CoAt_CIC-UNSW-NB15_Feeded-V2	CIC-UNSW-NB15	Fuzzers, Analysis, Reconnaissance	Scan
		Exploits, Backdoor, Shellcode, Worms	Worm
		Benign	Benign
	Distrinet-CIC-IDS2017	DDoS	DDoS
CoAt_CSE-CIC-IDS2018_Feeded	CSE-CIC-IDS2018	DDoS attacks-LOIC-HTTP, DDOS attack-HOIC, DDOS attack-LOIC-UDP	DDoS
		Infiltration, Bot	Worm
		Benign	Benign
	Distrinet-CIC-IDS2017	Portscan	Scan
CoAt_NF-UQ-NIDS-V2	NF-UQ-NIDS	scanning, Fuzzers, Reconnaissance, Analysis	Scan
		Infilteration, Exploits, Bot, Backdoor, Shellcode, Worm	Worm
		DDoS	DDoS
		Benign	Benign

PortScan, Reconnaissance, Fuzzers, and Analysis are closely tied to large stealthy scans because they all aim to gather critical information about a network while remaining undetected. Port scanning involves probing various ports on a network to identify which services are running, often using techniques designed to avoid triggering alarms [12]. Similarly, Reconnaissance activities focus on mapping out network structures and identifying vulnerabilities, often with minimal activity to stay under the radar [13]. Fuzzers work by sending unusual or unexpected inputs to systems, testing for weaknesses while blending into normal traffic patterns [14]. Analysis is an attack method where attackers gather and examine information through techniques such as traffic, cryptographic, code, data, and protocol analysis to identify and exploit system vulnerabilities for malicious purposes [8]. Together, these techniques allow attackers to quietly scan and assess networks without drawing attention. Therefore, we assigned a new label, “Scan,” in the “New Label” column of Table 5 to traffic originally labeled as PortScan, Reconnaissance, Fuzzers, and Analysis in the “Label of Extracted Data” column.

Infiltration, Exploits, Generic, Backdoor, Shellcode, Analysis, Bot, and Theft are key activities associated with worms because they represent various techniques that worms use to spread and gain control over systems. Worms typically exploit vulnerabilities (Exploits, Shellcode) to infiltrate networks and systems (Infiltration), where they establish persistent access through backdoors (Backdoor) [15,16,17]. Once inside, worms often convert infected systems into bots (Bot), which can be remotely controlled to participate in further attacks or spread the infection. These activities are part of a worm's automated nature, allowing it to propagate without direct human intervention [18]. Worms may also engage in theft (Theft) of sensitive data from compromised systems [19]. Overall, these behaviors highlight the self-replicating and destructive capabilities of worms as they navigate, exploit, and spread across networks. Therefore, we assigned the label “Worm” in the “New Label” column of Table 5 to traffic originally labeled as Infiltration, Exploits, Backdoor, Shellcode, Bot, and Theft in the “Label of Extracted Data” column.

We labeled traffic containing DDoS attacks with the label “DDoS” and traffic representing normal behavior with the label “Benign.” Unlike other categories, the majority of labels for DDoS attacks and normal traffic did not require additional grouping, as they were already consistent across the dataset. The only adjustments made were formatting changes to ensure uniformity, such as standardizing capitalization by converting labels to either uppercase or lowercase as needed. This approach ensured clarity and consistency in the dataset while preserving the original intent of the labels.

**Final Result**: The final step involves saving the transformed dataset in Parquet format, enabling efficient storage and retrieval of structured data. This format is particularly suitable for handling large datasets like CoAt-Set, facilitating rapid access and processing in collaborative research settings where scalability is essential.

### 4.4 Dataset folder structure overview

**Fig. 4:**
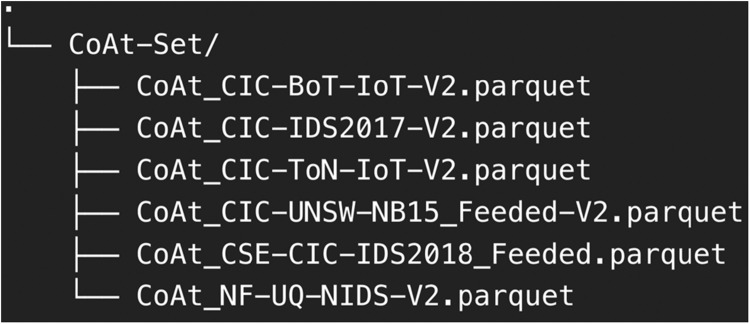
CoAt-Set folder snapshot.

The CoAt-Set dataset is structured into six Parquet files after the preprocessing and transformation processes, with each file representing a distinct dataset as illustrated in Fig. 4. The files include CoAt_CIC-BoT-IoT-V2, CoAt_CIC-IDS2017-V2, CoAt_CIC-ToN-IoT-V2, CoAt_CIC-UNSW-NB15_Feeded-V2, CoAt_CSE-CIC-IDS2018_Feeded, and CoAt_NF-UQ-NIDS-V2. This organization allows researchers to easily access and utilize datasets tailored to specific simulation needs, supporting a wide range of experiments in IDS research. The choice of the Parquet file format ensures efficient data storage and processing, enhancing usability across various machine learning platforms.

The majority of the datasets in CoAt-Set, such as those derived from CIC-BoT-IoT, CIC-IDS2017, CIC-ToN-IoT, CIC-UNSW-NB15, and CSE-CIC-IDS2018, are based on CICFlowMeter features. These datasets are particularly suited for simulating multi-sensor CIDS in heterogeneous networks. By integrating traffic data from diverse network environments, CoAt-Set enables researchers to explore collaborative detection strategies that account for different types of network traffic and attack patterns. This setup is critical for advancing distributed and multi-layered intrusion detection capabilities in complex, real-world network scenarios.

In addition to its support for multi-sensor simulations, CoAt-Set includes the CoAt_NF-UQ-NIDS-V2 dataset, which is uniquely designed for Non-IID scenario simulations. This dataset, built with NetFlow features, is derived from a unified set of multiple public sources and represents the heterogeneity often encountered in real-world network traffic. It allows researchers to simulate and study the challenges posed by varying traffic distributions across nodes, making it an invaluable resource for developing and evaluating IDS techniques in Non-IID environments. Together, these datasets provide a comprehensive platform for addressing key challenges in CIDS research.

### 4.5 Dataset transformation code

The details code to process the dataset can be seen in this link: 10.5281/zenodo.14279865.

## Limitations

While the CoAt-Set dataset provides valuable insights into coordinated attack patterns, there are limitations associated with its development through dataset transformation techniques. Transformed data may not fully capture the complexity and unpredictability of real-world cyberattacks. The lack of randomness present in real attacks could lead to detection algorithms that perform poorly in actual scenarios. Additionally, models trained on transformed data risk overfitting to specific patterns, limiting their effectiveness against unseen threats.

The dataset's derivation from multiple sources introduces potential inconsistencies in data quality and labeling, which can affect the uniformity and reliability of the dataset. Variations in how data is collected, preprocessed, and labeled across sources may introduce biases or inaccuracies, further complicating model training and validation. Challenges inherent to dataset transformation include the potential loss of realism, as transformation processes may simplify or alter the data in ways that reduce its ability to accurately represent real-world behaviors. Biases introduced during transformation can further skew model training, and validating models without real-world data is difficult, raising concerns about their practical performance [20].

To address these limitations, future work could integrate real-world attack data to refine and validate the transformed models. Additionally, standardizing preprocessing and labeling practices across sources can mitigate inconsistencies, ensuring a more robust and reliable dataset. By overcoming these challenges, researchers can enhance the dataset's relevance, improving detection strategies and contributing to a deeper understanding of coordinated attacks in the ever-evolving cybersecurity landscape.

## Ethics statement

The authors affirm that they have complied with the ethical standards for publication in Data in Brief. They also confirm that this study does not involve human participants, animal research, or any data gathered from social media platforms.

## Credit author statement

**Aulia Arif Wardana:** Conceptualization, Methodology, Investigation, Writing – original draft; **Grzegorz Kołaczek:** Supervision, Conceptualization, Methodology; **Parman Sukarno:** Writing – review & editing.

## Data Availability

Mendeley DataCoAt-Set (Coordinated Attack Dataset) on Heterogeneous Computer Network (Original data).
